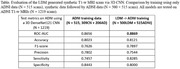# Generative AI improves MRI‐based Detection of Alzheimer’s Disease by using Latent Diffusion Models and Convolutional Neural Networks

**DOI:** 10.1002/alz.089958

**Published:** 2025-01-09

**Authors:** Nikhil J Dhinagar, Sophia I Thomopoulos, Paul M. Thompson

**Affiliations:** ^1^ Imaging Genetics Center, Mark and Mary Stevens Neuroimaging and Informatics Institute, Keck School of Medicine, University of Southern California, Marina del Rey, CA USA

## Abstract

**Background:**

As new treatments (such as the anti‐amyloid vaccine, lecanamab) emerge for Alzheimer’s disease (AD) and other dementias, approaches are required to rapidly diagnose AD at the earliest possible stage, and to assess disease progression and prognosis. In January 2024, the FDA approved the first AI tool to predict AD progression based on magnetic resonance imaging (MRI) [1]. Here we train a generative AI approach based on latent diffusion models ‐ to encode disease effects on brain structures. We show how AI‐based generation of synthetic scans can complement existing datasets for AD‐related diagnostic tasks.

**Method:**

We analyzed 4,098 3D T1‐weighted brain MRI scans (556M/632F, 55.7 ‐ 92.8 years) from the Alzheimer’s Disease Neuroimaging Initiative (ADNI). We trained a latent diffusion model (LDM) [2] as our generative AI framework. The model was conditioned on the disease status of AD versus healthy controls to generate synthetic brain MRI scans. For validation, the generated synthetic MRI scans was used to initialize a 3D DenseNet‐121 CNN (convolutional neural network) [3], and then further fine‐tuned for AD classification.

**Result:**

The table below summarizes the AD classification performance of a 3D CNN on a test set of 1,219 scans from ADNI. The synthetic data improved performance by over 2%, i.e., receiver‐operator characteristic curve‐area under the curve (ROC‐AUC) from 0.8656 to 0.8869.

**Conclusion:**

In this work, we trained a latent diffusion model to generate brain MRI scans. When these scans were used to train a deep neural network to detect AD, performance increased. Future work will test these generative AI models for interpretable disease detection via counterfactual image generation, and to discover factors that affect AD onset and progression via meta‐data encoding in the generative model.

**References**:

[1]. J. Shugart, FDA Approves BrainSee, AI Software That Purportedly Predicts AD, AlzForum News, available at: https://www.alzforum.org/news/research‐news/fda‐approves‐brainsee‐ai‐software‐purportedly‐predicts‐ad, 2024.

[2] W. H. L. Pinaya et al., “Brain Imaging Generation with Latent Diffusion Models,” MICCAI Workshop on Deep Generative Models (DGM4MICCAI) 2022, pp 117‐126.

[3] N. Dhinagar et al., “Video and Synthetic MRI Pre‐training of 3D Vision Architectures for Neuroimage Analysis,” SPIE Medical Imaging 2024.